# Examining the influence of ultraviolet C irradiation on recombinant human γD-crystallin

**Published:** 2010-12-16

**Authors:** Steven S.-S. Wang, Wen-Sing Wen

**Affiliations:** Department of Chemical Engineering, National Taiwan University, Taipei, Taiwan

## Abstract

**Purpose:**

Human γD crystallin is a principal protein component of the human eye lens and associated with the development of juvenile and mature-onset cataracts. Exposure to ultraviolet (UV) light is thought to perturb protein structure and eventually lead to aggregation. This work is aimed at exploring the effects of UV-C irradiation on recombinant human γD-crystallin (HGDC).

**Methods:**

Recombinant HGDC proteins were expressed in *E. coli* strain BL21(DE3) harboring plasmid pEHisHGDC and purified using chromatographic methods. The proteins were then exposed to UV-C light (λ_max_=254 nm, 15 W) at the intensity of 420, 800, or 1850 μW/cm^2^. The UV-C-unexposed, supernatant fraction of UV-C-exposed, and re-dissolved precipitated fraction of UV-C exposed preparations were characterized by SDS–PAGE, turbidity measurement, CD spectroscopy, tryptophan fluorescence spectroscopy, acrylamide fluorescence quenching analysis, and sulfhydryl group measurements.

**Results:**

The turbidity of the HGDC sample solution was found to be positively correlated with HGDC concentration, UV-C irradiation intensity, and UV-C irradiation duration. When exposed to UV-C, HGDC sample solutions became visibly turbid and a noticeable amount of larger protein particle, perceptible to the naked eye, was observed upon prolonged irradiation. The precipitated fraction of irradiated HGDC sample was found to be re-dissolved by guanidine hydrochloride. Electrophoresis, acrylamide fluorescence quenching, and spectroscopic analyses revealed differences in structures among the non-irradiated HGDC, the supernatant fraction of irradiated HGDC, and the re-dissolved precipitated fraction of irradiated HGDC. Through the use of L-cysteine, the measurements of sulfhydryl contents, and the reducing as well as non-reducing SDS–PAGE, our data further suggested that disulfide bond formation and/or cleavage probably play an important role in aggregation and/or precipitation of HGDC elicited by UV-C irradiation.

**Conclusions:**

Our findings highlight the close connections among disulfide bond cleavage and/or formation, intermolecular interactions, and the resultant formation of aggregates of HGDC induced by UV-C irradiation. The results from this research may not only contribute to the understanding of the environmental factors causing protein aggregation but also have implications for deciphering the molecular mechanism of cataractogenesis.

## Introduction

Human age-onset cataract disease, defined as the light scattering opacification of the crystallin lens in the eye, is a common eye disease that affects populations worldwide. Crystallins are water-soluble structural proteins in the vertebrate eye lens. They are divided into three categories: α-, β-, and γ-crystallins. The high concentration and short-range order of these crystallins within the lens play important roles in maintaining lens transparency. As the lens ages, modifications or damage, such as photooxidation, deamidation, disulfide bond formation [1,2], and cleavage [3], result in incorrect interactions, unfolding, oligomerization, and aggregation of proteins. Such events can lead to disruption of the arrangement of crystallins and the development of opacities known as cataracts.

Human γD-crystallin (HGDC) is a 173-residue monomeric eye lens protein synthesized during embryonic development that must remain soluble in the anucleated cells of the adult human eye lens for proper vision [4,5]. The high-resolution crystal structure of HGDC has been resolved by X-ray diffraction [6]. Structurally, HGDC is composed of duplicated Greek-key motifs that form two intercalated, anti-parallel β-sheets, which comprise the NH_2_- and COOH-terminal domains. The two domains of HGDC contain two tryptophans that have been investigated to determine the mechanisms of unfolding and refolding using fluorescence spectroscopy [7]. Evidence suggests that mutations in γD-crystallin are associated with juvenile-onset congenital cataracts in humans, further implicating HGDC in cataractogenesis [8,9]. Moreover, earlier studies have reported that several cases of cataracts resulted from mutations in HGDC. The R14C mutation leads to the formation of oligomers and causes juvenile-onset punctuate cataracts [5,10]. The R58H and R36S mutations result in reduced HGDC protein solubility [6,8,11]. The P23T mutation leads to decreased protein solubility and has no apparent significant effect on the overall protein structure in comparison to that of the native protein [11,12].

Ultraviolet (UV) light is believed to exert an impact on proteins and to induce damage on cells [13,14]. Moreover, UV light exposure is considered to be one of the environmental factors involved in lens cataractogenesis during aging [15]. UV-C is a shortwave UV irradiation (λ_max_=254 nm) and belongs to the major wavelengths in the UV spectrum. UV-C irradiation is the most biologically damaging range of solar radiation [16]. Several researchers have reported that UV irradiation has an adverse impact on proteins, and several hypotheses accounting for the interaction(s) have put forth: involvement in the generation of free radicals or reactive oxygen species, or modification of protein structures [17-19]. The detailed interacting mechanisms, however, remain largely unknown. The conformational and functional consequences of UV-C irradiation have already been demonstrated for a variety of proteins, but not for HGDC [20,21].

In this research, with UV-C irradiation used as a structural perturbant, we set out to examine the effects of UV light exposure on the conformational changes of recombinant HGDC. We observed that UV-C irradiated recombinant HGDC solution became turbid and the formation of larger protein particles, perceptible to the naked eye, occurred upon prolonged (≥30 min) UV-C irradiation. We further compared the structural features of the supernatant and precipitated parts of the irradiated HGDC sample against that of the monomeric HGDC, using spectroscopic techniques and acrylamide fluorescence quenching analysis. Our results showed that the recombinant HGDC underwent changes in both secondary and tertiary structures upon UV-C irradiation. Moreover, with the aid of the measurement of sulfhydryl group content and the use of reducing agent L-cysteine, our data strongly suggested the involvement of disulfide bond formation and/or cleavage in HGDC aggregation induced by UV-C irradiation. The outcome from this work may contribute to deciphering the molecular mechanism of cataractogenesis.

## Methods

### Chemicals and materials

Salts, tryptone, yeast extract, and chromatography columns were purchased from Sigma-Aldrich (St Louis, MO). Kanamycin, imidazole, and isopropyl β-D-thiolgalactorpyranoside (IPTG) were obtained from Biobasic (Markham, ON, Canada). EZ Ni-agarose 6 resin was obtained from Lamda Biotech (St Louis, MO). All other chemicals were of reagent grade and obtained from Sigma unless otherwise specified.

### Cloning, expression and purification of HGDC protein

The 6×His-HGDC gene fragment from plasmid pQE1 [22] was amplified by polymerase chain reaction (PCR) using primers that introduced NdeI and BamHI restriction sites (forward primer and reverse primer are 5′-GAG GAG AAA TTA ACA TAT GAA ACA TCA CCA TCA-3′ and 5′-GCT TTG TTA GCA GCC GGA TCC AAA TTA AGA A-3′, respectively). The resultant purified PCR products were ligated to the resultant digested plasmid pET30b(+), resulting in the generation of pEHisHγDC. pEHisHγDC was transformed into the *E. coli* strain BL21(DE3), which was used to express the HGDC protein.

In a typical experiment, a single colony of *E. coli* strain BL21(DE3) harboring plasmid pEHisHGDC was inoculated in 50 ml LB medium (10% tryptone, 5% yeast extract, 10% NaCl) containing the appropriate antibiotic (kanamycin, 30 μg/ml) and grown with shaking at 200 rpm at 37 °C. After being induced by the addition of IPTG, the cultures were harvested by centrifugation and the pellets were suspended in lysis buffer (50 mM NaH_2_PO_4_, 5 mM imidazole, 300 mM NaCl, pH 8.0). The cell suspension was disrupted by ultrasonication and the insoluble materials were removed by centrifugation. The supernatant was passed through a Supelco liquid chromatography column (Sigma-Aldrich) and EZ Ni-agarose 6 resin (Lamda Biotech). The purified recombinant protein solution was dialyzed against 5 mM sodium phosphate buffer (pH 7.0) and stored at room temperature.

### Protein concentration determination and sodium dodecyl sulfate polyacrylamide gel electrophoresis (SDS–PAGE)

The protein concentrations of HGDC sample solutions were determined by the bicinchoninic acid assay (BCA) using bovine serium albumin as a standard [23]. A 50 μl aliquot of the resultant solution was mixed with 10 μl of 6× SDS–PAGE sample buffer (0.35 M Tris-HCl, pH 6.8, 10% sodium dodecyl sulfate, 36% glycerol, 0.012% bromophenol blue, with or without 5% of β-mercaptoethanol) and resolved by 15% SDS–PAGE. To estimate the percentage of cross-linking protein in UV-C irradiated solutions, images of destained gels were captured with a digital camera, and their corresponding pixel values were determined by the semi-quantitative SDS–PAGE method [24,25]. All images were analyzed by using Image-J software provided by the National Institute of Health.

### UV-C irradiation and turbidity measurement

All irradiation experiments were performed in a 1 cm light-path quartz cuvette. Solutions of HGDC in 5 mM sodium phosphate buffer (pH 7.0) were prepared and irradiated for a variety of time periods up to 60 min with a UV-C lamp (λ_max_=254 nm, 15 W). Irradiance set at 420, 800, or 1850 μW/cm^2^ was corrected by Goldilux MP series Ultraviolet probes, Model GCP-1 (Measuring Instruments Technology, Pretoria, Gauteng, South Africa). The irradiated solutions were diluted fivefold with 5 mM sodium phosphate buffer (pH 7.0) and their turbidities were measured by monitoring the absorption due to light scattering at a wavelength of 360 nm [26] using a Spectronic Genesys 5 Spectrophotometer (Artisan Scientific Corporation, Champaign, IL). Three measurements were performed, and the mean and standard deviation were obtained.

### Re-dissolution/re-solubilization of aggregation protein

In irradiation experiments, the UV-C-induced precipitates were harvested by centrifugation at 2,991× g for 2 min. The pellets were dissolved in various concentrations of guanidine hydrochloride (GdnHCl) ranging from 0 to 4.0 M and incubated at room temperature for 24 h. After incubation, the solutions were again centrifuged at 2,991× g for 2 min and the resultant supernatant solutions were used as re-dissolved, UV-C irradiated, precipitated HGDC samples for the following experiments.

### Circular dichroism (CD) spectroscopy

The CD spectra of non-irradiated, supernatant fraction of UV-C irradiated, and re-dissolved precipitated UV-C irradiated HGDC samples (0.2 mg/ml) with various concentrations of GdnHCl were collected over the wavelength range of 210–260 nm using a 0.1 cm quartz cell and a JASCO J-815 spectrometer (Jasco International Co., Ltd., Hachioji, Tokyo, Japan). Three scans each of duplicate samples were measured and averaged. Control buffer scans were run in duplicate, averaged, and then subtracted from the sample spectra. All experiments were performed at room temperature. The results have been plotted as ellipticity (mdeg) versus wavelength (nm).

### Tryptophan fluorescence spectroscopy

Tryptophan fluorescence intensities of HGDC sample solutions, including non-irradiated, supernatant fraction of UV-C irradiated, and re-dissolved precipitated UV-C irradiated HGDC samples at 0.2 mg/ml were recorded with a F-2500 Fluorescence Spectrophotometer (Hitachi, Tokyo, Japan) using excitation and emission wavelengths of 295 and 300–400 nm.

### Acrylamide fluorescence quenching experiments

GdnHCl and acrylamide stock solutions were prepared at 5 M in 5 mM sodium phosphate buffer (pH 7.0). The protein samples (non-irradiated, supernatant, and re-dissolved precipitated HGDC) were first mixed with GdnHCl and acrylamide stock solutions (final [protein]=0.2 mg/ml, final [GdnHCl]=0, 1.0, 1.5, and 2.0 M, final [acrylamide]=0, 0.1, 0.2, 0.3, and 0.4 M) and fluorescence measurements of the above mixtures were conducted using excitation at 295 nm and recording the fluorescence emission spectra from 300 to 400 nm on a F-2500 Fluorescence Spectrophotometer (Hitachi). The fluorescence quenching data (fluorescence emission intensity at 350 nm versus acrylamide concentration) were analyzed by using the Stern-Volmer Equation [27] listed as follows:

$$\frac{F_{0}}{F}=1+K_{sv}[Q]$$

where F_0_ was the fluorescence intensity in the absence of acrylamide quencher, F was the fluorescence intensity after addition of acrylamide quencher, and [Q] was the concentration of acrylamide quencher. A plot of F_0_/F versus [Q] yields a straight line with a slope of K_sv_, called the Stern-Volmer constant.

### Size exclusion chromatography (SEC)

The UV-C irradiated HGDC sample solutions taken at different exposure times were first mixed with the denaturant (SDS) and reducing agent (β-mercaptoethanol), passed through 0.22 μm filter units (nylon, 13 mm, 0.22 μm, Pall Life Sciences, Ann Arbor, MI), and subjected to size exclusion chromatography (SEC) analysis. The filtrate (1 ml) was injected into a Superdex^TM^ 75 10/300GL (GE Healthcare, Piscataway, NJ) previously equilibrated with the buffer solution (5 mM sodium phosphate buffer with 2% SDS and 10 mM β-mercaptoethanol, pH 7.0) and eluted with 0.25 ml/min mobile phase flow rate at room temperature. The corresponding chromatogram was recorded with the detector setting of wavelength at 280 nm.

### Quantification of protein thiols or sulfhydryl groups

The total concentration of sulfhydryl groups in HGDC samples was estimated according to the method using the thiol reagent 5,5′-dithiobis (2-nitro-benzoic acid) (DTNB) [28,29]. After being incubated in 1% SDS, aliquots (200 μl) of SDS-treated HGDC sample solutions, with or without UV-C irradiation, were mixed with 800 μl DTNB solution (1.1 mM, pH 7.0, 1% SDS) for 30 min at room temperature and then the absorbance at 412 nm (for DTNB) was measured on a μQuant microplate reader (Bio-Tek Instruments, Inc., Winooski, VT). The calibration curve was obtained using various dilutions of a 2.0 mM L-cysteine solution.

### Statistical analysis

The significance of the results was determined using a Student’s *t*-test on n independent measurements, where n is specified in the figure legend. Unless otherwise indicated, the significance was taken as p<0.05.

## Results

### Aggregation of HGDC induced by UV-C irradiation

**Figure 1:**
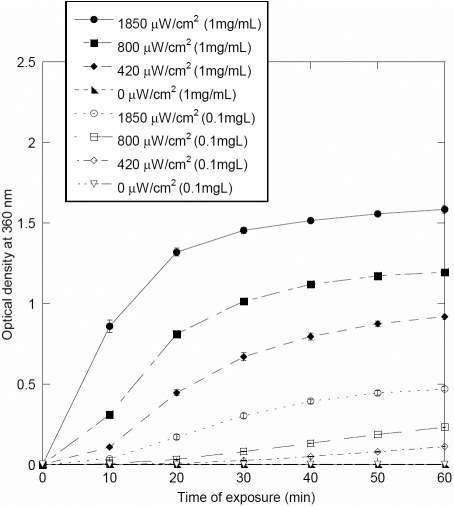
Effects of UV-C irradiation on the turbidity profile of HGDC at 1 mg/ml and 0.1 mg/ml. The doses of UV-C irradiation used were 0, 420, 800, and 1,850 μW/cm^2^.

The extent of HGDC aggregation as a function of UV-C exposure time and HGDC concentration was evaluated by measuring the turbidity (absorbance at 360 nm) of HGDC samples; the corresponding aggregation profiles are depicted in Figure 1. In the absence of UV-C irradiation (intensity=0 μW/cm^2^), HGDC samples showed negligible absorbance at 360 nm, indicating that aggregated species were not being produced. However, regardless of the UV-C irradiation intensity used, the turbidity of HGDC samples increased dramatically at first and then reached the plateau value, suggesting the formation of aggregates in the irradiated sample solutions. As expected, increasing irradiation time and/or higher HGDC concentration led to an elevation in the turbidity, and thereby, the level of aggregation. For example, at 1850 μW/cm^2^, the absorbances of 0.1 mg/ml HGDC were found to be 0.038±0.007 and 0.471±0.013 at 10 and 60 min, respectively, whereas absorbances of 1.0 mg/ml HGDC were 0.860±0.039 and 1.586±0.026 at 10 and 60 min, respectively. Moreover, HGDC irradiated at 1850 μW/cm^2^ was more turbid than that at 420 or 800 μW/cm^2^. Evidently, the turbidity of the HGDC sample solution was found to be positively correlated with HGDC concentration, UV-C irradiation intensity, and UV-C irradiation duration.

The physical appearance of the HGDC samples, under various experimental conditions, was monitored during the course of UV-C irradiation. When irradiated at 1850 μW/cm^2^, the HGDC sample solutions became visibly turbid upon UV-C irradiation and a noticeable amount of larger protein particle, perceptible to the naked eye, was observed in the solution after 30-min irradiation; precipitation was observed at the bottom of the tubes after ~40 min of irradiation. The precipitation was found to occur later at smaller doses of UV-C irradiation.

### Size distribution and degree of polymerization of HGDC induced by UV-C irradiation

**Figure 2:**
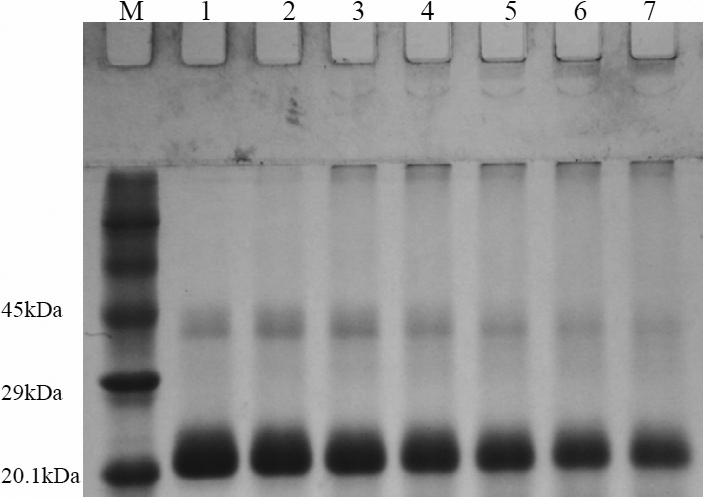
Non-reducing (without β-mercaptoethanol) SDS–PAGE analysis of UV-C irradiated HGDC samples (1 mg/ml) at different exposure times (M: protein marker, Lane 1: 0 min, Lane 2: 10 min, Lane 3: 20 min, Lane 4: 30 min, Lane 5: 40 min, Lane 6: 50 min, Lane 7: 60 min). The dose of UV-C irradiation used was 1,850 μW/cm^2^.

To identify the size distribution of species obtained by exposure of HGDC to UV-C irradiation, the irradiated (at 1,850 μW/cm^2^) HGDC samples at 1.0 mg/ml, taken at different times (0, 10, 20, 30, 40, 50, and 60 min), were electrophoresed on SDS–PAGE under non-reducing conditions (without β-mercaptoethanol). We demonstrate in Figure 2 that a prominent band at ~21 kDa, belonging to the monomeric species, and a slightly vague (a faint) band, corresponding to the dimeric species (see Figure 2, lane 1), were detected for the HGDC sample at t=0. When subjected to UV-C irradiation, the HGDC samples showed evidence of aggregated species, probably the dimer and oligomer, that migrated with molecular weights greater than ~42 kDa on the gel (e.g.: 20 min, see Figure 2, lane 3). Upon further irradiation, the bands corresponding to the aforesaid aggregated species became more apparent and a certain amount of macromolecular species was visibly trapped in the wells of the stacking gel (e.g.: 20 ~60 min, see Figure 2, lane 3–7). Moreover, as shown in Figure 2, lanes 2–3, some of the HGDC monomers were converted into HGDC dimeric species at the early stage of UV-C irradiation (e.g., ~20 min). However, as revealed by lanes 4–7 of Figure 2, further exposure to UV-C irradiation led to a significant reduction in the amount of HGDC dimers, which is most likely attributed to the involvement of dimeric species in the oligomerization or polymerization process.

By measuring the amount of unconverted HGDC that remained as monomeric species, we next sought to evaluate quantitatively the percentage of HGDC polymerization, the fraction of HGDC protein which was converted into the species with molecular weight/mass higher than ~42 kDa (corresponding to the dimeric HGDC speceis), as a function of irradiation time. As listed in Table 1, for example, after 20 or 50 min of irradiation, the percentage of HGDC polymerization was determined to be approximately 17.60% or 51.33%, respectively. Lengthened UV-C irradiation apparently gave rise to an increase in the percentage of HGDC polymerization.

**Table 1:** The percentage of HGDC polymerization obtained from non-reducing (without β-mercaptoethanol; β-ME) and reducing (with β-mercaptoethanol; β-ME) SDS–PAGE at various UV-C exposure times.

**Time (min)**	**0**	**10**	**20**	**30**	**40**	**50**	**60**
HGDC polymerization (%)^a^ without β-ME	0.00	7.31±1.88	17.60±6.23	30.64±5.57	41.95±6.12	51.33±6.35	53.64±7.73
HGDC polymerization (%)^a^ with β-ME	0.00	6.66±1.93	15.01±1.65	25.37±4.27	29.84±5.07	30.14±6.76	31.54±8.09

^a^Percentage of HGDC polymerization = the (Total amount of monomeric and dimeric HGDC at time 0) – (Total amount of monomeric and dimeric HGDC at time t/Total amount of monomeric and dimeric HGDC at time 0)

### Re-dissolution/re-solubilization of UV-C induced HGDC precipitates

**Figure 3:**
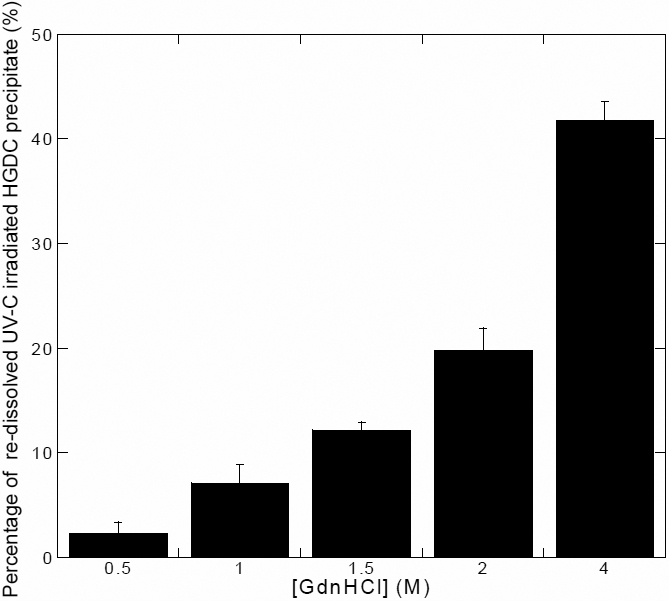
The percentage of re-dissolved UV-C irradiated precipitated HGDC sample as a function of GdnHCl concentration. The concentrations of GdnHCl used for precipitate re-dissolution were 0.5, 1.0, 1.5, 2.0, and 4.0 M.

To characterize the structural features of the species within the observed precipitates induced by UV-C irradiation, aliquots of precipitated species formed at the bottom of the tubes, taken at 60 min, were centrifuged and re-solubilized/re-suspended using GdnHCl. First, we wanted to know the effect of GdnHCl concentration on the quantity of HGDC that was re-solubilized from the precipitates. The fractions of HGDC protein present in the supernatant after GdnHCl re-solubilization were determined and depicted in Figure 3. Low levels (<2.5%) of the UV-C induced precipitates were only minimally dissolved in 0–0.5 M GdnHCl. Our results indicated that, upon further increase in GdnHCl concentration (>0.5 M), an increasingly greater extent of precipitate re-solubilization occurred. For instance, ~20% and ~42% of HGDC re-solubilized were perceived at 2.0 and 4.0 M of GdnHCl, respectively. It should be noted that, while the amount of precipitated protein that was re-dissolved was observed to be positively correlated with the concentration of GdnHCl used, given that treatment with higher concentrations of GdnHCl results in the increased probability of destruction of protein conformation/structure, knowledge of the influence of GdnHCl concentration on the structural changes of native HGDC would be absolutely critical in determining the optimal GdnHCl concentration for re-dissolution.

### Effect of UV-C on the secondary structure of HGDC

**Figure 4:**
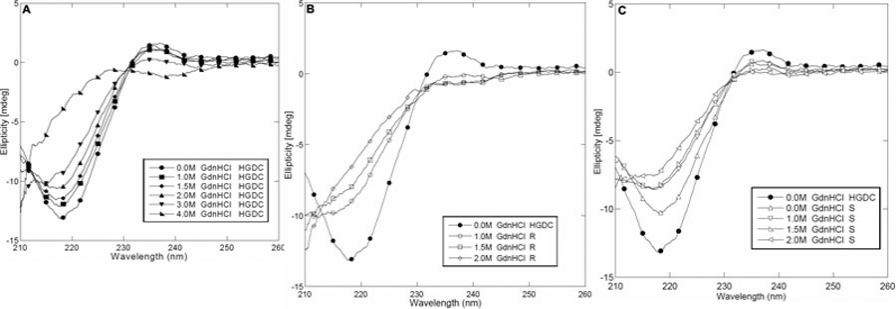
Representative far-UV CD spectra of HGDC samples with various concentrations of GdnHCl. **A**: Non-irradiated HGDC, **B**: Re-dissolved precipitated fraction of UV-C irradiated HGDC (R), and C: Supernatant fraction of UV-C irradiated HGDC (S).

To determine whether UV-C irradiation led to the changes in HGDC structure, circular dichroism spectroscopy was undertaken to analyze the secondary structures of the non-irradiated HGDC sample (native conformation), irradiated HGDC soluble sample, and UV-C irradiation-induced HGDC precipitate in the presence of various concentrations of GdnHCl. As Figure 4A attests, native HGDC shows a predominance of β-sheet secondary structures, as manifested by the far-UV CD spectrum with absorption minimum at ~218 nm, which is in agreement with the published result [5,22]. Since CD requires samples free of particulate matter and the UV-C irradiation-induced precipitate could be re-dissolved by GdnHCl, the CD measurements of the resultant soluble form of the above-mentioned precipitate became possible. The appropriate GdnHCl concentration used for re-dissolution was examined by comparing the far-UV CD spectra of the native HGDC with different concentrations of GdnHCl. Our results demonstrated that the samples of native HGDC, upon addition of GdnHCl up to 1.5 M, showed fairly similar far-UV CD spectra, suggesting that GdnHCl below 2.0 M had a negligible effect on the secondary structure of native HGDC and 1.5 M GdnHCl could be chosen for precipitate re-dissolution. On the contrary, the far-UV CD spectra in Figure 4B, for HGDC precipitated samples that were re-dissolved with various GdnHCl concentrations, demonstrate that the secondary structure of precipitated HGDC samples was significantly altered upon prolonged (e.g.: 60 min) UV-C irradiation at 1,850 μW/cm^2^. The extent of β-sheet structure in the UV-C irradiated samples was greatly reduced, as revealed by a significant increase in the negative ellipticity of the CD spectrum.

For comparison purposes, we also explored how GdnHCl concentration affected the structure of supernatant samples of the irradiated HGDC. As illustrated in Figure 4C, exposure to 2.0 M GdnHCl yielded a pronounced loss in the secondary structure, relative to the cases of 1.0 M and 1.5 M GdnHCl.

### Effect of UV-C irradiation on the tertiary structure of HGDC

**Figure 5:**
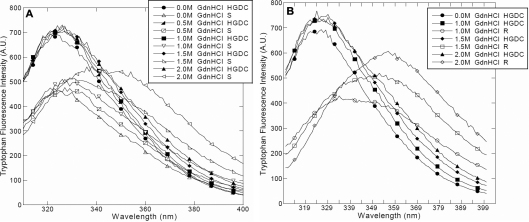
Representative tryptophan fluorescence spectra of HGDC samples with various concentrations of GdnHCl. **A**: Non-irradiated HGDC and supernatant fraction of UV-C irradiated HGDC (S). **B**: Non-irradiated HGDC and re-dissolved precipitated fraction of UV-C irradiated HGDC (R).

Tryptophan fluorescence of protein, mostly owing to the high sensitivity of fluorescence to tryptophans in their microenvironments, has been widely used in research work on ligand binding, folding-unfolding, protein conformational changes [30-32]. To further gain insights into the effect of UV-C irradiation on the conformational changes in HGDC, the tryptophan fluorescence spectra of aforesaid HGDC samples were also recorded. We show in Figure 5A that the peak fluorescence emission of non-irradiated HGDC samples in the presence of 0–2.0 M GdnHCl occurred at ~324–328 nm, which is in accordance with the data previously reported for the wild-type HGDC [5,22]. It is evident that not much difference in the tryptophan fluorescence spectra was detected among the HGDC samples (with and without GdnHCl).

As Figure 5A attests, in comparison with the non-irradiated counterpart, while negligible shifts in the wavelengths of emission maximum (λ_max_) were detected, lengthened exposure to UV-C irradiation (e.g.: 60 min) brought about a marked decrease in the intensity of the fluorescence emission spectra of the supernatant fraction of irradiated HGDC samples. Given its insoluble nature, we were not able to directly obtain the tryptophan fluorescence spectra from the irradiated precipitated HGDC samples. However, apart from the considerable reduction in fluorescence intensity, a pronounced red-shift in λ_max_ was observed in the fluorescence spectra of the re-suspended precipitated HGDC samples after 60 min of UV-C irradiation (shown in Figure 5B), implying that more solvent-exposed hydrophobic regions were obtained upon UV-C irradiation. Moreover, we found that λ_max_ of the supernatant or the re-dissolved precipitated HGDC sample was shifted toward a longer wavelength when an increasing concentration of GdnHCl was added, displaying a positive correlation between the red-shift in λ_max_ and the concentration of GdnHCl.

According to the preceding CD and fluorescence spectroscopy results, we could conclude that exposure of HGDC to UV-C irradiation brought about the structure perturbing effect. Our findings also clearly indicated that, as compared with its supernatant counterpart, the structure/conformation of the precipitated UV-C irradiated HGDC species was more susceptible to the addition of GdnHCl.

### Acrylamide fluorescence quenching of HGDC samples

Acrylamide, a polar, neutral molecule, can interact with all surfaces, independently of their charge, in the aqueous phase. We were interested in exploring how UV-C exposure affected the accessibility of HGDC’s tryptophan residues (Trp) to quencher through fluorescence quenching studies. Quenching of tryptophanyl fluorescence in proteins by addition of acrylamide can also be described by the Stern-Volmer plot, showing the dependence of the F_0_/F ratio of fluorescence intensity over the fluorescence intensity without quencher as a function of acrylamide concentration [27,33]. On increasing acrylamide concentration, we have observed a decrease in fluorescence intensity.

As revealed by the Stern–Volmer plots constructed for the samples of native (non-irradiated) HGDC and supernatant, as well as re-dissolved precipitated irradiated HGDC with varying concentrations of GdnHCl, there existed a rather linear relationship (R^2^>0.98) between the F_0_/F ratio and the acrylamide concentration in all cases (data not shown). Using the linear least-squares fitting of the data from acrylamide fluorescence quenching experiments to the Stern-Volmer equation (as described in the Methods section), the slopes or the Stern-Volmer constants (K_sv_) for the above-mentioned samples were determined and listed in Table 2. As expected, a higher concentration of GdnHCl brought an increase in the magnitude of K_sv_ for the respective sample. Moreover, it is apparent that, as opposed to the case of supernatant irradiated HGDC, a marked increase in the magnitude of K_sv_, from non-irradiated to the re-dissolved precipitated HGDC, was observed, which is indicative of a larger overall accessibility of Trp to acrylamide molecules in the re-dissolved precipitated irradiated HGDC, as compared with that of the supernatant irradiated fraction or the native form. Our fluorescence quenching results suggested that the non-irradiated, irradiated supernatant, and re-dissolved irradiated precipitate HGDC samples differed in the microenvironment of Trp or the degree of Trp exposure to surrounding medium.

**Table 2:** The summary of Stern-Volmer quenching constants (Ksv) of HGDC samples under different conditions.

**HGDC sample**	**K_sv_**
Non-irradiated HGDC	1.02
UV-C irradiated supernatant HGDC	1.24
Non-irradiated HGDC with 1.0 M GdnHCl	1.27
UV-C irradiated supernatant HGDC with 1.0 M GdnHCl	1.93
UV-C irradiated precipitated HGDC re-dissolved with 1.0 M GdnHCl	4.78
Non-irradiated HGDC with 1.5 M GdnHCl	1.48
UV-C irradiated supernatant HGDC with 1.5 M GdnHCl	2.64
UV-C irradiated precipitated HGDC re-dissolved with 1.5 M GdnHCl	6.33
Non-irradiated HGDC with 2.0 M GdnHCl	1.91
UV-C irradiated supernatant HGDC with 2.0 M GdnHCl	3.91
UV-C irradiated precipitated HGDC re-dissolved with 2.0 M GdnHCl	8.74

### Involvement of disulfide bonding in the HGDC structure induced by UV-C irradiation

**Figure 6:**
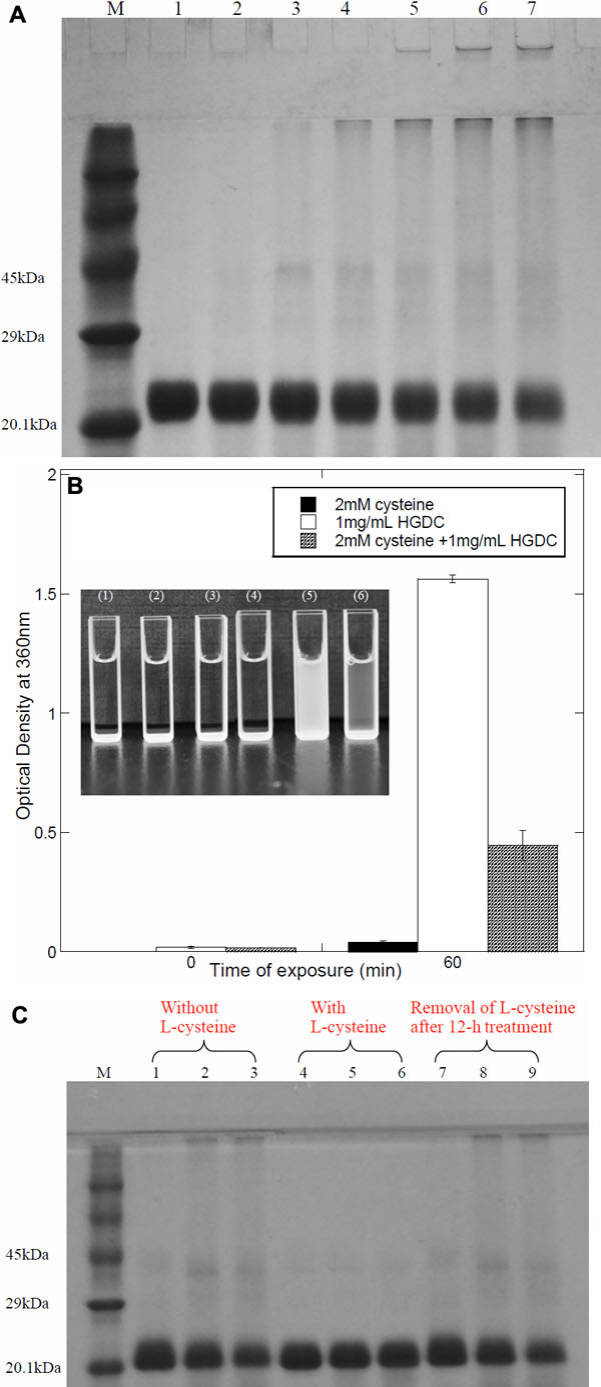
Effects of disulfide bonding on HGDC conformation induced by UV-C irradiation. **A**: Reducing (with β-mercaptoethanol) SDS-PAGE analysis of irradiated HGDC samples (1 mg/ml; M: protein marker, Lane 1: 0 min, Lane 2: 10 min, Lane 3: 20 min, Lane 4: 30 min, Lane 5: 40 min, Lane 6: 50 min, Lane 7: 60 min). **B**: Effects of L-cysteine on the turbidity of UV-C irradiated HGDC. The concentrations of L-cysteine and HGDC were 2 mM and 1 mg/ml. Inset: photograph of UV-C irradiated HGDC samples with and without L-cysteine (1): 2 mM L-cysteine without UV-C irradiation, (2): 1 mg/ml HGDC without UV-C irradiation, (3): mixture of 1 mg/ml HGDC and 2 mM L-cysteine without UV-C irradiation, (4): 2 mM L-cysteine with 60 min-UV-C irradiation, (5): 1 mg/ml HGDC with 60 min-UV-C irradiation, (6): mixture of 1 mg/ml HGDC and 2 mM L-cysteine with 60 min-UV-C irradiation). **C**: Non-reducing (without β-mercaptoethanol) SDS–PAGE analysis of 2 mM L-cysteine-HGDC mixture upon UV-C irradiation (M: protein marker, Lane 1: initial HGDC, Lane 2: UV-C irradiated HGDC (30 min-exposure), Lane 3: UV-C irradiated HGDC (60 min-exposure), Lane 4: initial 2 mM L-cysteine-HGDC mixture, Lane 5: UV-C irradiated 2 mM-L-cysteine-HGDC mixture (30 min-exposure), Lane 6: UV-C irradiated 2 mM-L-cysteine-HGDC mixture (60 min-exposure), Lane 7: L-cysteine-treated HGDC sample, Lane 8: 30 min UV-C exposed L-cysteine-treated HGDC sample, Lane 9: 60 min UV-C exposed L-cysteine-treated HGDC sample. L-cysteine-treated HGDC samples for Lanes 7–9 were obtained according to the following procedure: HGDC was first incubated with 2 mM L-cysteine for 12 h. The resultant L-cysteine-HGDC mixtures were then transferred to a Vivaspin 6 centrifugal concentrator filter unit with nominal molecular weight limit (NMWL) of 5 kDa (Sartorius, Gottingen, Germany). Followed by spinning at 4,307 rpm for 30 min, L-cysteine was removed from HGDC samples.

We were interested in understanding the possible correlation between disulfide bridges and structural changes in HGDC brought about by UV-C irradiation. To that end, reducing SDS–PAGE (with β-mercaptoethanol) analysis of HGDC samples with varying UV-C irradiation times was first conducted and the data are shown in Figure 6A. By comparing Figure 2 and Figure 6A, dissimilar patterns of molecular composition were noted on the gels run with and without reducing agent β-mercaptoethanol. Also, after performing the same calculation described previously, we found that the percentage of polymerization, in the case of reducing SDS–PAGE, was significantly lower than that for the non-reducing SDS–PAGE (shown in Table 1). The involvement of disulfide bond formation and/or cleavage was next investigated with the aid of the reducing agent L-cysteine, which was used to provide a more reducing incubation environment. We monitored the absorbance, at 360 nm, of HGDC samples in the presence and absence of L-cysteine following irradiation with UV-C. As depicted in Figure 6B, the absorbance of 2.0 mM L-cysteine-containing HGDC sample with 60-min UV-C irradiation was observed to be 0.45±0.06, which was reduced by ~71.5% over that of HGDC samples without L-cysteine. The photograph in the inset of Figure 6B further demonstrates that, upon 60-min irradiation, the HGDC sample with 2.0 mM L-cysteine was apparently less turbid to the naked eye than the sample without L-cysteine, suggesting superior suppression of aggregation/precipitation could be achieved when the reducing agent is added. Moreover, insight into the effect of L-cysteine on UV-C irradiated HGDC samples was obtained using SDS–PAGE analyses under non-reducing conditions. As revealed by lanes 4–6 of Figure 6C, HGDC co-incubated with 2.0 mM L-cysteine showed no indication of macromolecular species that migrated with molecular weights greater than ~42 kDa during the first 60 min of UV-C irradiation. We also conducted additional experiments, in which HGDC was treated with 2 mM L-cysteine for 12 h and exposed to UV-C irradiation after removing L-cysteine through centrifugation (detailed procedure see caption of Figure 6). Our results showed that, regardless of the UV-C irradiation duration, no noticeable difference was detected in the molecular profiles and turbidities between the HGDC sample and the HGDC sample with 12 h treatment of 2.0 mM L-cysteine, as revealed by lanes 7–9 in Figure 6C (turbidity data not shown). Finally, we directly measured the total sulfhydryl group (–SH) contents of HGDC samples via the reaction between thiol reagent DTNB and –SH groups. There was 88.26±4.91 μM (using DTNB) present in the 45 μM (1 mg/ml) HGDC sample before UV-C irradiation, which is close to the expected value (1 mol of HGDC has 2 moles of sulfhydryl groups). On the contrary, a substantially lower concentration of total –SH (17.09±6.79 μM using DTNB) was detected in the sample after 60 min of UV-C irradiation. Taken together, our preceding observations suggested that disulfide bond formation and/or cleavage probably play an important role in the mechanism of HGDC aggregation and/or precipitation elicited by UV-C irradiation.

## Discussion

Several previous studies have provided evidence that the structural and biochemical features of proteins can be affected by UV light. Exposure to UV irradiation could be correlated with the structural perturbation of proteins which might eventually lead to protein aggregation [17,34,35]. Moreover, the photo-oxidation of proteins induced by light exposure can result in various kinds of modifications, such as cross-linkages [36], fragmentation of covalent bonds, and changes in different amino acids [21,36,37]. Redecke and coworkers [35] have demonstrated that the conversion and aggregation of recombinant murine and human prion proteins were greatly influenced by UV-B irradiation. Damage of the serum amyloid P component, caused by exposure to UV-C irradiation, was identified through the use of two dimensional electrophoresis and mass spectrometry [38]. In a study with type I collagen, it was found that the serum amyloid P component’s fibril formation was affected by UV irradiation and that the fiber formed was structurally different from its intact counterpart [39]. Results from an earlier research revealed that, after UV-B irradiation, corneal aldehyde dehydrogenase experienced photodamage with a decrease in its enzymatic activity and changes in its surface hydrophobicity as well as hydrodynamic behavior but not in its secondary structure [40]. Thakur and Rao [34] found that the prion protein experienced structural changes and formed unordered or fibrillar aggregates when exposed to UV light of 290 nm. Moreover, it was shown that the denaturation of a main whey protein in bovine milk, α-lactoglobulin, by irradiation at 298 nm, brought about cleavage of disulfide bonds, leading to protein aggregation [28].

Others have reported that several eye-lens proteins are prone to aggregate upon exposure to UV light [21,41]. Eye-lens proteins experience variation in conformation or quaternary packing, giving to the opacity of the lens [42]. For example, the subunits of α-crystallin, αA-crystallin and αB-crystallin, respond differently to thermal denaturation, UV stress [43], and gamma-ray irradiation [44]. Also, γ-crystallins were documented to have lower susceptibility to photo-aggregation than that of β-crystallins [45]. Structural features of α-crystallin or αB-crystallin under UV-B irradiation were characterized using circular dichroism [46] and small-angle neutron diffraction [47], respectively. By monitoring the focal length variability and the morphology of various sectors, it was found that UV-A irradiation led to alterations in lens optical quality and damages to the surface properties of lens fiber cells [48]. The aggregation kinetics induced by UV radiation (at 308 nm) were delineated for the recombinant α-crystallin, β-crystallin, γ-crystallin, or their mixture of different species, using light scattering analysis [36,49].

Aside from the conformational alteration and aggregation, evidence has indicated that UV light exposure could bring about certain modifications in crystallin proteins. It was previously shown that the degradation of tryptophan residues and the attenuation of chaperone activity were detected in bovine lens α-crystallin upon exposure to UV-B irradiation (290–320 nm) [50]. As for the subunit A of bovine lens α-crystallin, a high level irradiation (e.g.: gamma irradiation) was found to oxidize tryptophan and methionine residues [51,52], and to isomerize aspartic acid residues [52], thus decreasing the chaperone activity. Analogous results were obtained when exposing bovine lens α-crystallin to UV-C irradiation [21]. By comparing the results obtained from recombinant βA3-crystallin and its NH_2_-terminus truncated derivative, Sergeev and coworkers [36] pointed out that, under UV-B irradiation, the augmented aggregation propensity is positively correlated with the absence of the NH_2_-terminal extension of βA3-crystallin. Also, the aggregation in the truncated βA3-crystallin was found to be associated with photolysis and oxidation of tryptophan and methionine residues [36].

While the aggregative behaviors induced by irradiation (e.g.: gamma ray, UV-A, UV-B, or UV-C) have been reported in several lens crystallins, crystallin subunits or their mixed populations from bovine species were used in most studies. To the best of our knowledge, however, no report was concerned with evaluating the influence of UV-C irradiation on HGDC. The presented research was aimed at delineating the structural changes and aggregating behavior of the purified recombinant wide-type HGDC upon UV-C irradiation. We first showed that the turbidity (absorbance at 360 nm) of recombinant HGDC samples increased following UV-C irradiation and a long exposure of HGDC to UV-C resulted in precipitation or formation of insoluble aggregates seen with the naked eye (e.g.: 30.64% of the HGDC protein was precipitated within 30 min at 1850 μW/cm^2^; Figure 1). Through SDS–PAGE electrophoresis analysis under the non-reducing condition, we then demonstrated that the UV-C irradiated HGDC samples had a considerable amount of higher molecular weight species accompanied with a decrease in the level of monomer, which was not seen in the non-irradiated control (Figure 2). In addition, the ratio of total amount of monomeric and dimeric species of the irradiated sample to that of the non-irradiated control (or percentage of HGDC polymerization) was found to be inversely (or positively) correlated with irradiation time (Figure 2 and Table 1). Our findings apparently indicated that UV-C illumination can give rise to the formation of HGDC aggregates.

To spectroscopically compare the structures of protein species present in the HGDC samples, re-dissolution of HGDC precipitate resulting from UV-C irradiation was conducted. With its protein-denaturing ability, GdnHCl at different concentrations were tested (Figure 3 and Figure 4A) and 1.5 M GdnHCl was determined to be the optimal concentration for precipitate re-suspension/re-solubilization. The CD spectra of irradiated HGDC (supernatant fraction and precipitated fraction) samples in the presence of varying GdnHCl concentrations were monitored (Figure 4B,C). Our CD spectra also indicated that exposing HGDC to UV-C irradiation shifted the protein secondary structure away from predominantly β-sheet conformation detected in the native conformation (non-irradiated) of HGDC (Figure 4C).

As revealed by tryptophan fluorescence spectroscopy and acrylamide quenching analysis, the UV-C irradiation brought about alterations in the tertiary structure of HGDC samples, which were manifested as a reduced tryptophan fluorescence emission and a red shift in tryptophan fluorescence emission spectra or an increased exposure of hydrophobic regions to solvent (Figure 5A,B) and a larger Stern-Volmer constant or an enhanced accessibility of Trp residues to the quenchers (Table 2). Our findings evidently indicated that the conformation (secondary or tertiary structures) of HGDC was markedly affected by UV-C exposure. Also, closer inspection of our data showed that the re-dissolved precipitated fraction of HGDC was slightly more sensitive to the denaturant (GdnHCl) than its supernatant fraction (Figure 5A,B). These results are in line with the ones reported previously from the study using bovine γ-crystallin [27].

With regards to the possibility of forming disulfide-linked aggregates or precipitates upon UV-C irradiation, several experiments were conducted and several points could be made according to our observations: (1) It is reasonable to presume that the buffer system used for non-reducing SDS–PAGE disperses noncovalently linked protein aggregates into monomers whereas aggregated species linked through disulfide bridges tended to remain intact. Also, the use of reducing condition would lead to the destruction of disulfide bonds. Dissimilar SDS–PAGE patterns of molecular compositions were obtained on separating the irradiated samples under the non-reducing and reducing conditions (Figure 2 and Figure 6A) and higher percentages of polymerization were derived from the non-reducing SDS–PAGE case (Table 1). (2) A significant reduction in turbidity or aggregation of HGDC samples was observed when L-cysteine was added in the HGDC sample (Figures 6B). (3) As revealed by SDS–PAGE analysis, comparing 2 mM L-cysteine-containing HGDC samples with the HGDC control sample showed no precipitates with high molecular masses were found in the well of the stacking gel during UV-C irradiation (Figure 6C). Also, upon removal of L-cysteine through centrifugation (detailed procedure see legend of Figure 6), a negligible difference was observed in the molecular distributions between the 2 ml L-cysteine treated HGDC sample and the HGDC control (Figure 6C). The above-mentioned experimental findings evidently suggested that the reducing environment certainly prevents the species of higher molecular masses from forming (or polymerization) in UV-C exposed HGDC samples. (4) A direct quantitation of the −SH group contents of HGDC samples with and without UV-C irradiation revealed that a considerable decrease in −SH content was detected in the irradiated sample as compared with its non-irradiated counterpart.

**Figure 7:**
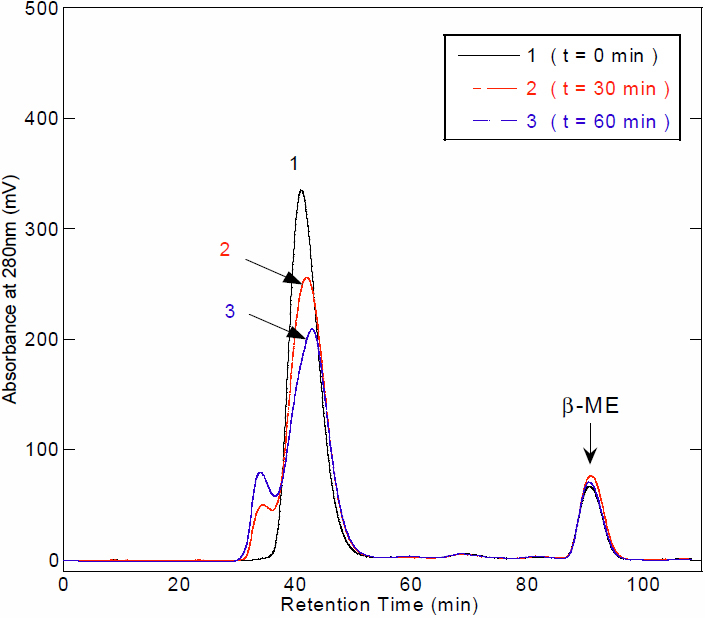
The size exclusion chromatograms of HGDC samples with and without UV-C irradiation. All protein samples were denatured by mixing 2% SDS and 10 mM β-mercaptoethanol (β-ME). The collected HGDC samples were first passed through 0.22 μm filter units, and then subjected to size exclusion chromatography (SEC) analysis. The operating conditions of SEC analysis used were: injection volume, 1 ml; flow rate of mobile phase, 0.25 ml/min; detector, UV at 280 nm; column, Superdex™ 75 10/300GL (GE Healthcare); mobile phase composition, 5 mM sodium phosphate, 2% SDS, 10 mM β-ME (pH 7.0). Chromatogram 1: HGDC without UV-C irradiation (exposure time=0); chromatogram 2: HGDC with 30 min-UV-C irradiation; chromatogram 3: HGDC with 60 min-UV-C irradiation. The peak that was observed to elute at ~91 min was verified to correspond to β-ME according to the control experiment (chromatogram of β-ME alone not shown).

We were also interested in knowing if the degradation of HGDC sample occurred upon UV-C irradiation. To that end, we have performed additional experiments, in which UV-C irradiated HGDC protein solutions taken at different exposure times were first mixed with the denaturant (SDS) and reducing agent (β-mercaptoethanol), passed through 0.22 μm filter units, and subjected to size exclusion chromatography (SEC) analysis. The control solutions of monomeric HGDC sample and β-mercaptoethanol were first injected into SEC separately. After recording the resultant chromatograms for monomeric HGDC (shown in Figure 7) and β-mercaptoethanol (chromatogram not shown), the corresponding retention times were identified to be ~41 and ~91 min, respectively. Assuming that UV-C irradiation leads to the fragmentation or degradation of HGDC or the generation of shortened HGDC peptides, the presence of peaks located between those of the native HGDC and β-mercaptoethanol would be detected. As depicted in Figure 7, a relatively smaller peak was observed to elute a little earlier than the major peak at ~41 min which corresponds to the monomeric species of HGDC. The earlier small peak that increased with UV-C exposure time (with a concurrent decrease in the major peak) is likely to be ascribed to the formation of dimeric and/or oligomeric HGDC species. Moreover, no distinguishable peaks were detected within the range of ~41–91 min in the chromatogram. Therefore, we can conclude from our experimental finding that HGDC protein is not likely to be degraded or fragmented when subjected to UV-C irradiation for up to ~60 min. Despite the fact that some evidences suggest that, in addition to protein aggregation, the breakdown or cleavage of polypeptide chains may also be a possible chemical change caused by radiation [53,54], the results obtained from this work demonstrate otherwise for HGDC, at least under our experimental condition of up to 60 min UV-C irradiation.

Taken together, our findings undoubtedly highlight the close association among disulfide bond cleavage/formation, intermolecular interactions, and the resultant formation of aggregates of HGDC induced by UV-C irradiation. While further analysis of the details of the mechanism is warranted and additional experiments are underway, we believe the results from this research may not only contribute to the understanding of the environmental factors causing protein aggregation and but also have implications for deciphering the molecular mechanism of cataractogenesis.

## References

[1] Hanson SRA Smith DL Smith JB Deamidation and disulfide bonding in human lens gamma-crystallins. Exp Eye Res 1998 67 301 12 9778411 10.1006/exer.1998.0530

[2] Stephan DA Gillanders E Vanderveen D Freas-Lutz D Wistow G Baxevanis AD Robbins CM VanAuken A Quesenberry MI Bailey-Wilson J Juo SHH Trent JM Smith L Brownstein MJ Progressive juvenile-onset punctate cataracts caused by mutation of the gamma D-crystallin gene. Proc Natl Acad Sci USA 1999 96 1008 12 9927684 10.1073/pnas.96.3.1008 PMC15341

[3] Srivastava OP Srivastava K Degradation of gamma D- and gamma s-crystallins in human lenses. Biochem Biophys Res Commun 1998 253 288 94 9878530 10.1006/bbrc.1998.9728

[4] Meakin SO Breitman ML Tsui LC Structural and evolutionary relationships among 5 members of the human gamma-crystallin gene family. Mol Cell Biol 1985 5 1408 14 4033658 10.1128/mcb.5.6.1408-1414.1985 PMC366871

[5] Pande A Pande J Asherie N Lomakin A Ogun O King JA Lubsen NH Walton D Benedek GB Molecular basis of a progressive juvenile-onset hereditary cataract. Proc Natl Acad Sci USA 2000 97 1993 8 10688888 10.1073/pnas.040554397 PMC15742

[6] Basak A Bateman O Slingsby C Pande A Asherie N Ogun O Benedek GB Pande J High-resolution X-ray crystal structures of human gammaD crystallin (1.25 A) and the R58H mutant (1.15 A) associated with aculeiform cataract. J Mol Biol 2003 328 1137 47 12729747 10.1016/s0022-2836(03)00375-9

[7] Flaugh SL Kosinski-Collins MS King J Interdomain side-chain interactions in human gamma D crystallin influencing folding and stability. Protein Sci 2005 14 2030 43 16046626 10.1110/ps.051460505 PMC2279314

[8] Pande A Pande J Asherie N Lomakin A Ogun O King J Benedek GB Crystal cataracts: human genetic cataract caused by protein crystallization. Proc Natl Acad Sci USA 2001 98 6116 20 11371638 10.1073/pnas.101124798 PMC33431

[9] Santhiya ST Manohar MS Rawlley D Vijayalakshmi P Namperumalsamy P Gopinath PM Loster J Graw J Novel mutations in the gamma-crystallin genes cause autosomal dominant congenital cataracts. J Med Genet 2002 39 352 8 12011157 10.1136/jmg.39.5.352 PMC1735119

[10] Guthalugu NK Balaraman M Kadimi US Optimization of enzymatic hydrolysis of triglycerides in soy deodorized distillate with supercritical carbon dioxide. Biochem Eng J 2006 29 220 6

[11] Pande A Annunziata O Asherie N Ogun O Benedek GB Pande J Decrease in protein solubility and cataract formation caused by the Pro23 to Thr mutation in human gamma D-crystallin. Biochemistry 2005 44 2491 500 15709761 10.1021/bi0479611

[12] Evans P Wyatt K Wistow GJ Bateman OA Wallace BA Slingsby C The P23T cataract mutation causes loss of solubility of folded gamma D-crystallin. J Mol Biol 2004 343 435 44 15451671 10.1016/j.jmb.2004.08.050

[13] Krohne TU Hunt S Holz FG Effect of 308 nm excimer laser irradiation on retinal pigment epithelium cell viability in vitro. Br J Ophthalmol 2009 93 91 5 18952645 10.1136/bjo.2008.145102

[14] Janig E Haslbeck M Aigelsreiter A Braun N Unterthor D Wolf P Khaskhely NM Buchner J Denk H Zatloukal K Clusterin associates with altered elastic fibers in human photoaged skin and prevents elastin from ultraviolet-induced aggregation in vitro. Am J Pathol 2007 171 1474 82 17872975 10.2353/ajpath.2007.061064 PMC2043509

[15] Truscott RJW Age-related nuclear cataract - oxidation is the key. Exp Eye Res 2005 80 709 25 15862178 10.1016/j.exer.2004.12.007

[16] Kovécs G Fekete A Berces A Ronto G The effect of the short wavelength ultraviolet radiation. An extension of biological dosimetry to the UV-C range. J Photochem Photobiol B 2007 88 77 82 17604638 10.1016/j.jphotobiol.2007.02.001

[17] Mafia K Gupta R Kirk M Wilson L Srivastava OP Barnes S UV-A-induced structural and functional changes in human lens deamidated alpha B-crystallin. Mol Vis 2008 14 234 48 18334940 PMC2255029

[18] Kyselová Z Krizanova L Soltes L Stefek M Electrophoretic analysis of oxidatively modified eye lens proteins in vitro: implications for diabetic cataract. J Chromatogr A 2005 1084 95 100 16114241 10.1016/j.chroma.2004.10.001

[19] Bossi O Gartsbein M Leitges M Kuroki T Grossman S Tennenbaum T UV irradiation increases ROS production via PKC delta signaling in primary murine fibroblasts. J Cell Biochem 2008 105 194 207 18523985 10.1002/jcb.21817

[20] Kaminska A Kowalska M A study of the lens crystallin's photodegradation in the presence of beta-carotene. Polym Degrad Stabil 1999 66 9 15

[21] Fujii N Uchida H Saito T The damaging effect of UV-C irradiation on lens alpha-crystallin. Mol Vis 2004 10 814 20 15534584

[22] Kosinski-Collins MS Flaugh SL King J Probing folding and fluorescence quenching in human gammaD crystallin Greek key domains using triple tryptophan mutant proteins. Protein Sci 2004 13 2223 35 15273315 10.1110/ps.04627004 PMC2279819

[23] Smith PK Krohn RI Hermanson GT Mallia AK Gartner FH Provenzano MD Fujimoto EK Goeke NM Olson BJ Klenk DC Measurement of protein using bicinchoninic acid. Anal Biochem 1985 150 76 85 3843705 10.1016/0003-2697(85)90442-7

[24] Casserly U Mooney MT Troy D Standardisation and application of a semi-quantitative SDS-PAGE method for measurement of myofibrillar protein fragments in bovine longissimus muscle. Food Chem 2000 69 379 85

[25] Tomaszewska-Gras J Kijowski J Schreurs FJG Quantitative determination of titin and nebulin in poultry meat by SDS-PAGE with an internal standard. Meat Sci 2002 62 61 6 10.1016/s0309-1740(01)00228-5 22061192

[26] Ellozy AR Ceger P Wang RH Dillon J Effect of the UV modification of alpha-crystallin on its ability to suppress nonspecific aggregation. Photochem Photobiol 1996 64 344 8 8760574 10.1111/j.1751-1097.1996.tb02469.x

[27] Phillips SR Wilson LJ Borkman RF Acrylamide and iodide fluorescence quenching as a structural probe of tryptophan microenvironment in bovine lens crystallins. Curr Eye Res 1986 5 611 9 3757547 10.3109/02713688609015126

[28] Kehoe JJ Remondetto GE Subirade M Morris ER Brodkorb A Tryptophan-mediated denaturation of beta-lactoglobulin A by UV irradiation. J Agric Food Chem 2008 56 4720 5 18522413 10.1021/jf0733158

[29] Ellman GL Tissue sulfhydryl groups. Arch Biochem Biophys 1959 82 70 7 13650640 10.1016/0003-9861(59)90090-6

[30] Eftink MR Fluorescence techniques for studying protein-structure. Methods Biochem Anal 1991 35 127 205 2002770 10.1002/9780470110560.ch3

[31] Eftink MR The use of fluorescence methods to monitor unfolding transitions in proteins. Biochemistry (Mosc) 1998 63 276 84 9526124

[32] Di Stasio E Bizzarri P Misiti F Pavoni E Brancaccio A A fast and accurate procedure to collect and analyze unfolding fluorescence signal: the case of dystroglycan domains. Biophys Chem 2004 107 197 211 14962600 10.1016/j.bpc.2003.09.005

[33] Rezaei-Ghaleh N Ramshini H Ebrahim-Habibi A Moosavi-Movahedi AA Nemat-Gorgani M Thermal aggregation of alpha-chymotrypsin: Role of hydrophobic and electrostatic interactions. Biophys Chem 2008 132 23 32 17964060 10.1016/j.bpc.2007.10.001

[34] Thakur AK Rao CM UV-light exposed prion protein fails to form amyloid fibrils. PLoS ONE 2008 3 e2688 18628989 10.1371/journal.pone.0002688 PMC2442654

[35] Redecke L Binder S Elmallah MIY Broadbent R Tilkorn C Schulz B May P Goos A Eich A Rubhausen M Betzel C UV-light-induced conversion and aggregation of prion proteins. Free Radic Biol Med 2009 46 1353 61 19249347 10.1016/j.freeradbiomed.2009.02.013

[36] Sergeev YV Soustov LV Chelnokov EV Bityurin NM Backlund PS Wingfield PT Ostrovsky MA Hejtmancik JF Increased sensitivity of amino-arm truncated beta A3-crystallin to UV-light-induced photoaggregation. Invest Ophthalmol Vis Sci 2005 46 3263 73 16123428 10.1167/iovs.05-0112

[37] Dalsgaard TK Otzen D Nielsen JH Larsen LB Changes in structures of milk proteins upon photo-oxidation. J Agric Food Chem 2007 55 10968 76 18044829 10.1021/jf071948g

[38] Chan HL Gaffney PR Waterfield MD Anderle H Matthiessen HP Schwarz HP Turecek PL Timms JF Proteomic analysis of UVC irradiation-induced damage of plasma proteins: Serum amyloid P component as a major target of photolysis. FEBS Lett 2006 580 3229 36 16697377 10.1016/j.febslet.2006.05.002

[39] Menter JM Patta AM Sayre RM Dowdy J Willis I Effect of UV irradiation on type I collagen fibril formation in neutral collagen solutions. Photodermatol Photoimmunol Photomed 2001 17 114 20 11419538 10.1034/j.1600-0781.2001.170302.x

[40] Uma L Hariharan J Sharma Y Balasubramanian D Effect of UVB radiation on corneal aldehyde dehydrogenase. Curr Eye Res 1996 15 685 90 8670773 10.3109/02713689609008910

[41] Manzer R Pappa A Estey T Sladek N Carpenter JF Vasiliou V Ultraviolet radiation decreases expression and induces aggregation of corneal ALDH3A1. Chem Biol Interact 2003 143-144 45 53 12604188 10.1016/s0009-2797(02)00171-0

[42] Rao SC Rao CM Balasubramanian D The conformational status of a protein influences the aerobic photolysis of its tryptophan residues - melittin, beta-lactoglobulin and the crystallins. Photochem Photobiol 1990 51 357 62 2356231 10.1111/j.1751-1097.1990.tb01722.x

[43] Liao JH Lee JS Chiou SH Distinct roles of alpha A- and alpha B-crystallins under thermal and UV stresses. Biochem Biophys Res Commun 2002 295 854 61 12127973 10.1016/s0006-291x(02)00784-2

[44] Fujii N Nakamura T Sadakane Y Saito T. Differential susceptibility of alpha A- and alpha B-crystallin to gamma-ray irradiation. Biochim Biophys Acta 2007 1774 345 50 17258947 10.1016/j.bbapap.2006.12.001

[45] Hott JL Borkman RF Concentration-dependence of transmission losses in UV-laser irradiated bovine alpha-crystallin, beta-H-crystallin, beta-L-crystallin and gamma-crystallin solutions. Photochem Photobiol 1993 57 312 7 8451296 10.1111/j.1751-1097.1993.tb02293.x

[46] Lin SY Ho CJ Li MJ UV-B-induced secondary conformational changes in lens alpha-crystallin. J Photochem Photobiol B 1999 49 29 34 10365444 10.1016/S1011-1344(99)00010-X

[47] Sugiyama M Fujii N Morimoto Y Kurabayashi S Vigild ME Nakagawa T Sato T Itoh K Mori K Fukunaga T Structural evolution of human recombinant alpha B-crystallin under UV irradiation. Biomacromolecules 2008 9 431 4 18211002 10.1021/bm7004802

[48] Azzam N Levanon D Dovrat A Effects of UV-A irradiation on lens morphology and optics. Exp Gerontol 2004 39 139 46 14724074 10.1016/j.exger.2003.09.020

[49] Ostrovsky MA Sergeev YV Atkinson DB Soustov LV Hejtmancik JF Comparison of ultraviolet induced photo-kinetics for lens-derived and recombinant beta-crystallins. Mol Vis 2002 8 72 8 11951082

[50] Schauerte JA Gafni A Photodegradation of tryptophan residues and attenuation of molecular chaperone activity in alpha-crystallin are correlated. Biochem Biophys Res Commun 1995 212 900 5 7626128 10.1006/bbrc.1995.2054

[51] Finley EL Dillon J Crouch RK Schey KL Radiolysis-induced oxidation of bovine alpha-crystallin. Photochem Photobiol 1998 68 9 15 9679446

[52] Fujii N Hiroki K Matsumoto S Masuda K Inoue M Tanaka Y Awakura M Akaboshi M Correlation between the loss of the chaperone-like activity and the oxidation, isomerization and racemization of gamma-irradiated alpha-crystallin. Photochem Photobiol 2001 74 477 82 11594064 10.1562/0031-8655(2001)074<0477:cbtlot>2.0.co;2

[53] Voss P Hajimiragha H Engels M Ruhwiedel C Calles C Schroeder P Grune T Irradiation of GAPDH: a model for environmentally induced protein damage. Biol Chem 2007 388 583 92 17552905 10.1515/BC.2007.068

[54] Lee Y Song KB Effect of gamma-irradiation on the molecular properties of myoglobin. J Biochem Mol Biol 2002 35 590 4 12470593 10.5483/bmbrep.2002.35.6.590

